# Promoting prevention with economic arguments – The case of Finnish occupational health services

**DOI:** 10.1186/1471-2458-8-130

**Published:** 2008-04-22

**Authors:** Eila Kankaanpää, Aki Suhonen, Hannu Valtonen

**Affiliations:** 1 Research and Development in OHS, Institute of Occupational Health, POB 93, FIN-70701 Kuopio, Finland; 2 Department of Health Policy and Management, University of Kuopio, POB 1627, FIN-70211 Kuopio, Finland

## Abstract

**Background:**

Both social and ethical arguments have been used to support preventive occupational health services (OHS). During the 1990s it became more common to support political argumentation for occupational health and safety by converting the consequences of ill health at work into monetary units. In addition, OHS has been promoted as a profitable investment for companies, and this aspect has been used by OHS providers in their marketing.

Our intention was to study whether preventive occupational health services positively influence a company's economic performance.

**Methods:**

We combined the financial statements provided by Statistics Finland and employers' reimbursement applications for occupational health services (OHS) costs to the Social Insurance Institution. The data covered the years 1997, 1999 and 2001 and over 6000 companies. We applied linear regression analysis to assess whether preventive OHS had had a positive influence on the companies' economic performance after two or four years.

**Results:**

Resources invested in preventive OHS were not positively related to a company's economic performance. In fact, the total cost of preventive OHS per turnover was negatively correlated to economic performance.

**Conclusion:**

Even if OHS has no effect on the economic performance of companies, it may have other effects more specific to OHS. Therefore, we recommend that the evaluation of prevention in OHS should move towards outcome measures, such as sickness absence, disability pension and productivity, when applicable, both in occupational health service research and in practice at workplaces.

## Background

Both social and ethical arguments have been used to support preventive occupational health services (OHS). It has been regarded as a fundamental right of each worker to reach the highest attainable standard of health, and workers' health at work should be protected [1,2]. Prevention was perceived as valuable, at any rate better and cheaper than a cure [3], and therefore economic analyses were not required.

During the 1990s it became more common to reinforce political argumentation for occupational health and safety by converting the consequences of ill health at work into monetary units [4-6]. Moreover, OHS has been promoted as a profitable investment for companies [7,8], a viewpoint that has been used by OHS providers in their marketing.

Occupational health personnel have a role in assessing the health risks at the workplace – both environmental risks and problems in the functioning of the working community. They offer guidance on how to carry out interventions to improve working conditions and well-being at work and assist employees in maintaining their health. They also carry out interventions themselves, organize groups e.g. for persons with neck problems or obesity, participate in the planning and implementation of return-to-work policies, and act as facilitators in organizational development projects [9,10].

The interventions also have an economic dimension. Through improvement of working conditions, the costs of occupational accidents and diseases can be lowered. More importantly, these improvements can also lead to increased productivity [8,11]. If employees are motivated and committed to their work, they are willing to improve the services and products, which leads to higher customer satisfaction and faster payment of invoices. This reduces receivable accounts and thus provides higher return on capital employed. Employees' initiatives also concern internal processes. The result will be less rework and smooth processes that lower operating expenses [12]. The costs of sickness absence and disability pensions can be lowered with health related interventions [13], which has immediate positive financial effects for a company. In the long run, this will mean reductions in the company's health and insurance pension premiums due to lowered social security costs [8].

We study the assumption that if a company invests more in preventive occupational health services this would mean more interventions and, consequently, more favourable outcomes. Finally, there would be an impact on the profitability of the company.

We wanted to study the above described previously unexplored relationship between a company's economic performance and its investment in preventive occupational health services. The small amount of research in this area may be due to insufficient data. The circumstances in Finland, however, enable examination of this relationship, as it has been obligatory for employers to arrange preventive occupational health services for their employees since 1979. Preventive services include both individual and workplace activities. Employers can voluntarily organize GP level medical services, and they are entitled to reimbursement for the costs of preventive and medical services. Because of the reimbursement system, there is an employer-based register of the contents and costs of OHS. In this study, we combined this register with the firms' financial statements gained from Statistics Finland. Firm-specific identification codes were used in the processing of the financial statement data.

Our objective was to determine whether preventive occupational health services positively influence a company's economic performance.

## Methods

We examined the relationship between companies' investment in preventive OHS in 1997 and 1999 and the companies' economic performance four or two years later in 2001. We had the opportunity to use micro-level data from the companies.

Statistics Finland collects the financial statements of all Finnish firms from tax authorities. The register also contains data such as number of persons employed by the company, year of establishment, registered office, and industry.

The Social Insurance Institution (SII) registers employers' reimbursement applications for OHS. This register contains data on the service mix and the costs incurred. We chose to use registers from the years 1997, 1999 and 2001. In 1997, the reformed reimbursement system for the promotion of activities supporting work ability had been in force for two years. As this project was launched, 2001 was the last year for which all reimbursement applications had been processed. Companies apply for reimbursement within six months of closing their accounts, after which it takes over a year to process all the applications at the SII. We then merged this register with the Statistics Finland data, using firm-specific identification codes.

Finnish firms are a heterogeneous group. Table 1 presents the exclusion criteria. In 2001, 40% of a total of 226,000 firms were actually self-employed private persons and 15% had limited or unlimited liabilities. These and all other juridical forms except companies were excluded. Companies are defined as clearly for-profit organizations, and the legislation on bookkeeping and financial statements guarantees high quality of economic performance data. To enable assessment of whether the preceding investment in preventive OHS has had an impact on the company's economic performance, the companies had to have been in business continually through 1997–2001 (financial statements were available for this period).

**Table 1:** Exclusion criteria and number of companies in study

Number of firms in 2001	226 000
Number of companies in 2001	99 428
Financial statements for 1997, 1999 and 2001	64 597
Quality of the data rated good or excellent in 2001	32 522
Turnover > €50 000 for each of the three years	24 380
Personnel > 10 in 2001	7 013
Trimming of the key ratios for 2001 (1% both tails)	6 271

Statistics Finland has graded the quality of the financial statements into three categories. It only uses financial statements from firms when the quality of the data is graded excellent or good in its own publications and analysis. We used the same criteria, as in the third category many of the rows in the income statements have to be estimated.

The Act on Occupational Health Services applies only to firms that have employees. We assumed that a company's turnover had to exceed a certain level in order for it to be able to employ someone. With this in mind, we excluded companies with a turnover of less than €50,000 p.a. The number of employees in the Statistics Finland register also includes all short-term contracts, which might have been valid for a couple of hours only. Thus we left out companies with less than ten employees in 2001. By trimming both tails of all key ratios we were left with 6271 companies for the analysis.

After the exclusions, the number of companies fell from almost 100,000 to 6271. However, with view to average Finnish companies in 2001, the companies included in the study were rather typical in their location in terms of region and type of municipality. Regionally, the companies were mainly located in the south: 37% in Uusimaa, the region around the capital city of Helsinki, 36% in Southern Finland, 8% in Eastern Finland, 12% in Central Finland, 7% in Northern Finland, and less than 1% in Åland. Most of the companies were situated in urban municipalities (74%), and the rest were split between semi-urban (14%) and rural municipalities (12%).

The size distribution of the companies in the study naturally differed from that of all Finnish companies as those with less than ten employees were excluded.

The industry distribution of the included companies differed from that of total Finnish companies in three industries: the share of companies in real estate, renting, and business activities was smaller in the study population than in all Finnish companies (28%). These companies were small: 92% employed less than ten persons and were therefore excluded from the study. The financial intermediation industry disappeared completely, as this industry has special regulations concerning financial statements and cannot be compared with companies from other industries. The share of companies from the combined industry group of mining and quarrying plus manufacturing was higher than in all Finnish companies (14%). The size and industry distribution of the companies included in the study is presented in Additional file 1.

The average turnover of the companies in the study was about €24 million, and the average age of a company was 18 years.

Statistics Finland calculated the key ratios for all companies (Table 2). They are all derived from financial statements and commonly used in assessing companies' economic performance. We used the five key indicators for profitability as an outcome measure for company economic performance. Key indicators for industries differ [14], and there are also geographical and regional differences, mainly due to differences in competitiveness [15]. The size of the company is also a factor in economic performance [16].

**Table 2:** Key ratios of companies in 2001 (N = 6 271)

Key ratio	Mean	Median	Standard Deviation
Profitability			

Operating margin, % What is left over from the company's earnings after paying for variable costs of production divided by net sales.	10.03	8.73	8.29
Operating profit, % Earnings before interest and taxes (EBIT) divided by net sales.	6.5	5.67	7.15
Net result, % (Total revenues - total expenses) divided by net sales = Shows whether a company has earned or lost money with its business in the accounting period.	4.25	3.65	6.15
Total result, % Net result + extraordinary revenues - extraordinary expenses divided by net sales	4.05	3.27	5.76
Profit/loss for the accounting period, % The profit/loss result after tax payments divided by net sales.	4.11	3.25	5.57

Solidity			

Return on capital assets, % Shows how profitable the company is relative to its total assets. = Net income/total assets	14.05	12.43	13.59
Return on investment, % Evaluates the efficiency of an investment = (gain from investment - cost of investment)/cost of investment.	26.83	21.5	32.28
Return on equity, % Shows how much profit is made relative to the owners' investment in the company = Net income/shareholders equity	24.71	21.01	59.87
Relative indebtedness, % Company's liabilities divided by its turnover.	32.18	23.92	28.39
Less than 40%: Good			
40–80%: Satisfactory			
More than 80%: Poor			
Equity ratio, % The percentage of equities from the balance sheet	43.43	43.13	23.37
Over 40%: Good			
20–40%: Satisfactory			
Less than 20%: Poor			

Liquidity			

Quick Ratio Company's ability to meet its obligations.	0.51	0.23	0.73
Over 1: Good			
0.5–1: Satisfactory			
less than 0.5: Poor			
Current Ratio Company's ability to meet short term debt obligations.	0.54	0.38	0.61
Over 2: Good			
1–2: Satisfactory			
less than 1: Poor			

In Finland preventive occupational health services cover almost all employed persons; only in micro firms with less than 10 employees the employers has not always organized OHS services for the workplace. According to a population survey conducted in 2006, two out of three employees had attended an occupational health examination in the past three years, and around half of them had had occupational health personnel assessing their workplace in the past three years. Although organizing medical services is voluntary for employers, over 90% of employees can obtain GP level services from their OHS unit. Around half of the primary care level GP visits of these employees take place within OHS [17].

To be able to compare investment in preventive OHS between companies, we chose two different points of view: resources per employee and OHS's share of total costs per turnover (importance compared to other uses of resources in the company, comparable e.g. to costs of prevention per gross national product, GNP).

Because of the specific features of the Finnish reimbursement system, we measured the company's investment in OHS per employee both in monetary and temporal terms. Until 1995, the prices in municipal health centres were set by the State Council and did not cover the costs of providing these services [18]. Many municipal units have been slow in changing their pricing policy: in 2000 one in three were still using the regulated prices from 1994. In the companies' own OHS units, the costs of preventive and medical services in reimbursement applications are often divided according to the shares of maximum reimbursement (40% for prevention and 60% for medical services) and not according to the resources used.

The time variable was calculated from the SII register data. Workplace and group activities had originally been registered in hours. We converted the number of health examinations into minutes based on information from previous studies or an expert assessment of the contents of OH personnel's work in different provider models. All activities were summed up into the variable Occupational Health (OH) Personnel's Time per Employee. The time resource and costs correlated strongly in all other provider models (0.6–0.8), but not in the companies' own units. The price level in municipal OHS units was about 40% lower than in other provider models (euros per OH personnel minute). Thus time resource is a better measure for investment in preventive OHS for both the companies' own units and municipal OHS units. Therefore, the decision was made to leave the costs per employee out of the analysis.

On average, the 6721 companies invested in preventive OHS €39.50 per employee in 1997 and €46.00 in 1999. This sum bought the companies some 22 minutes of OH personnel time per employee for each of the two years. Among the companies who had applied for reimbursement, the costs were the highest in the companies' own OHS units (€91 for prevention and €138 for medical services per employee) and lowest in municipal health centres (prevention €62 and medical services €42 per employee).

The average share of total costs for preventive OHS per turnover was 0.04%.

We assumed that the provider model could have an impact on company performance. The companies' own units are generally believed to be able to integrate their activities more efficiently into the company than other providers. However, in this study, the OHS provider model had no effect on the key ratios and was thus excluded from the models.

The connection between investment in OHS and the companies' economic performance was analyzed using linear regression analysis. The investment in preventive occupational health services was not dependent on the company's previous economic success. The correlations between the investment in 2001 and the key ratios in 1997 or 1997 were all small (absolute values were less than 0.1).

We tested the models using the regression specification error test (RESET test). It can be used for testing the functional form of a model, especially to detect non-linearities and omitted variables [19]. RESET test revealed that the relationship between dependent and some independent variables was logarithmic rather than linear. The models were also tested for multicollinearity.

We used two different software packages in the analysis. The SAS software package was used in excluding and recoding and the STATA for the analysis.

## Results

### Model

The five key indicators of the profitability of the company were the dependent variables, each in its turn. The independent variables in the model were investment in preventive OHS either in minutes per employee (log) or as the total cost of prevention per turnover (%), all in 1997 or in 1999. Therefore, we conducted twenty regression analyses to study the connection between investment in preventive OHS and company profitability.

The other independent variables were the company's past economic performance (equity ratio in 1999, with higher equity ratio indicating greater opportunity to make profitable investments), size of the company (log number of employees in 2001, log turnover in 1999), and age of the company.

Some of the confounding variables were dummies, and the coefficients are meaningful only when compared to the reference group. Industry was included in the model because key ratios differ according to industry; in this study the reference group was wholesale and retail trade. Geographical regions were included to represent booming or declining regional economies (6 counties in Finland, reference region Uusimaa). The type of municipality is an indicator of the size of the local market, for both the company's products and for OHS. Municipalities were classified into three groups: city, semi-urban, and rural.

We checked the correlations between independent variables (see Additional file 2), and found no multicollinearity.

### Company's economic performance

Operating profit represents here the economic performance of the company in 2001. Table 3 includes two different models for operating profit, one for each indicator of the company's investment in preventive occupational health services in 1997.

**Table 3:** Regression models for operating profit in 2001, investment in preventive OHS measured with two variables

	Dependent: operating profit in 2001			
	Coefficient		Coefficient	

Constant	7.439	***	8.040	***
Preventive OHS in 1997				
Minutes per employee (log)	-0.050			
Total costs/turnover			-349.552	*
Equity ratio in 1999	0.060	***	0.060	***
Equity ratio in 1999*2	0.000380	***	0.000381	***
Log number of employees in 2001	-0.080		-0.020	
Log turnover in 1999	-0.348	**	-0.401	**
Age of company	-0.018	*	-0.018	*
Industry				
Agriculture, hunting and forestry, fishing	3.875	***	3.888	***
Mining and quarrying, manufacturing	2.216	***	2.249	***
Electricity, gas, and water supply	0.788		0.781	
Construction	2.081	***	2.107	***
Hotels and restaurants	1.482	**	1.507	**
Transport, storage, and communication	1.109	**	1.109	**
Real estate, renting, and business activities	2.448	***	2.494	***
Education	-3.739	*	-3.710	*
Health and social work	2.910	***	2.984	***
Other community, social and personal service activities	-0.436		-0.406	
Reference: Wholesale and retail trade				
Region				
South	0.119		0.114	
East	0.181		0.178	
Central	-0.109		-0.118	
North	0.590		0.582	
Åland	0.884		0.882	
Ref. Uusimaa				
Municipality				
Semi-urban	0.198		0.182	
Rural	0.199		0.180	
Ref. city				
Adjusted R^2^	0.12		0.12	
Reset F(3, 6244)	5.10	p = 0.0016	5.25	p = 0.0013

* p < 0.05, ** p < 0.01, ***p < 0.001

The preceding investment in preventive OHS measured as OH personnel time per employee in 1997 had a negative coefficient which was statistically non-significant.

When the share of total preventive OHS costs per turnover represented investment, the coefficient was negative and statistically significant.

The results of the models for other key indicators were very similar to those presented in Table 3. All coefficients for time per employee variables were non-significant, and negative in 9 out of 10 models. For the costs per turnover variable, the coefficient was negative in all ten models, and statistically significant in nine out of ten models.

Success seems to follow success: a higher equity ratio in 1999 was connected with better key indicators for profitability in 2001. The age of the company and turnover were negatively related to profitability but no correlation was found between profitability and the geographical or regional location of the company.

The adjusted R^2^s were low in general, highest in the models for operating margin (18%), and about 12% for other key indicators of profitability.

## Discussion

In this study, we could not find support for the hypothesis that a company's investment in preventive OHS would have a positive effect on the company's profitability after two or four years. The coefficients for the variables representing preceding investment in preventive OHS in the regression models were negative in almost all models. Those for OH personnel time per employee variable were always non-significant. When the investment was measured as the total costs of preventive OHS as a share of turnover, the coefficients were all negative, and statistically significant in nine out of ten models.

The power of the study and the follow-up time were sufficient to yield significant relevant results. In Finland, we had the unique opportunity of combining economic indicators from companies with data on OHS.

Economic performance of a company is a complex phenomenon, and difficult to decipher exhaustively with this kind of data. The explanatory power of the regression model for the economic performance of the company was low, but not deviant from other studies with similar design explaining a firm's economic performance [20,21]. Some independent variables that might affect economic performance were lacking, which was also shown in the RESET tests. Had we had information on matters such as management, marketing and research and development, the explanatory power of the models would have been higher. However, if these omitted variables are uncorrelated with our key variables (as tested by RESET test, in fact), the results relevant for our study questions are unaffected by these omitted variables.

From a company's point of view and compared to other investments in intangibles (16% of the turnover), such as R&D and marketing, and investments in tangibles (13% of the turnover), such as equipment, the investment in OHS is minimal, less than 0.5% of turnover on average [22]. The economic performance of a company is much more dependent on other factors than preventive occupational services, and as an outcome measure economic performance is too distant to actually correlate with preventive OHS. If one wants to show that occupational health services are profitable for the company cost-benefit and cost-effectiveness analysis should be used. Good examples of such studies are Tompa et al. and Taimela et al. [13,23]. In addition, occupational health services units can be an economical way to provide medical curative care [24].

A line of argumentation based on a viewpoint quite separate from profitability for the company could also be used to advocate the importance of prevention. Sometimes prevention can be valuable from the viewpoint of society even though it would not be profitable for the company. Legislation [25] is one way to impose obligations for employers to avoid negative externalities to the society and the employees, i.e. the costs of ill health due to work. To promote the consumption of preventive services, the society can subsidize employers: the reimbursement system in Finland is one example of this.

## Conclusion

The conclusion of our study is that there is no evidence to support the positive effect of investment in preventive OHS on the economic performance of a company.

However, it cannot be concluded that preventive OHS has no positive effects. We would rather recommend that all prevention would be judged on it success in achieving its specific objectives that are related to its core activities. The discussion as to whether OHS is beneficial should move towards more specific outcomes, such as sickness absence, disability pension and productivity, when applicable, both in occupational health service research and in practice at workplaces. What is not effective cannot be profitable for the company either.

In general, it might be quite difficult to prove that prevention has some impact on the economy (see [3], p. 13–23). This means the discussion about the relationship between health and productivity, or the wealth of a nation, will continue [26] within occupational health. However the first step, the link between prevention and health should be given priority when planning new research on the area.

## Competing interests

The authors declare that they have no competing interests.

## Authors' contributions

EK and HV planned the study. AK carried out the analysis under supervision by HV and EK. EK drafted the manuscript, and the other authors have read and approved it.

## Pre-publication history

The pre-publication history for this paper can be accessed here:



## Supplementary Material

Additional file 1 Companies by number of employees and industry in 2001. Click here for file

Additional file 2 Correlations of variables. Click here for file
