# Supplementary material for: Promoting prevention with economic arguments – The case of Finnish occupational health services
Source: BMC Public Health. 2008 Apr 22;8:130. doi: 10.1186/1471-2458-8-130 (PMC2386811; doi:10.1186/1471-2458-8-130)
Supplement: Additional file 1 — Companies by number of employees and industry in 2001. [file 1471-2458-8-130-S1.pdf]

Additional file 1 – Companies by number of employees and industry in 2001

Number of companies<br>(per cent by row)<br>[per cent by column]	Industry										
Number of employees	AB	CD	E	F	G	H	I	K	M	N	O	Total
10-19	32<br>(1.5)<br>[66.7]	554<br>(25.8)<br>[24.0]	16<br>(0.7)<br>[21.3]	375<br>(17.4)<br>[42.5]	523<br>(24.3)<br>[38.5]	79<br>(3.7)<br>[34.5]	199<br>(9.3)<br>[44.2]	272<br>(12.7)<br>[38.2]	3<br>(0.1)<br>[21.4]	54<br>(2.5)<br>[61.4]	44<br>(2.1)<br>[42.7]	2151<br><br>[34.3]
20-99	15<br>(0.5)<br>[31.3]	1287<br>(40.3)<br>[55.7]	48<br>(1.5)<br>[64.0]	437<br>(13.7)<br>[49.5]	687<br>(21.5)<br>[50.6]	125<br>(3.9)<br>[54.6]	175<br>(5.5)<br>[38.9]	339<br>(10.6)<br>[47.6]	9<br>(0.3)<br>[64.3]	25<br>(0.8)<br>[28.4]	45<br>(1.4)<br>[43.7]	3192<br><br>[50.9]
100-499	1<br>(0.1)<br>[2.1]	370<br>(49.8)<br>[16.0]	10<br>(1.4)<br>[13.3]	59<br>(7.9)<br>[6.7]	117<br>(15.8)<br>[8.6]	21<br>(2.8)<br>[9.2]	61<br>(8.2)<br>[13.6]	88<br>(11.8)<br>[12.3]	1<br>(0.1)<br>[7.1]	5<br>(0.7)<br>[5.7]	10<br>(1.4)<br>[9.7]	743<br><br>[11.8]
500-	0<br>(0)<br>[0]	98<br>(53.0)<br>[4.2]	1<br>(0.5)<br>[1.3]	12<br>(6.5)<br>[1.4]	32<br>(17.3)<br>[2.4]	4<br>(2.2)<br>[1.8]	15<br>(8.1)<br>[3.3]	14<br>(7.6)<br>[2.0]	1<br>(0.5)<br>[7.1]	4<br>(2.2)<br>[4.6]	4<br>(2.2)<br>[3.9]	185<br><br>[3.0]
Total	48<br>(0.8)	2309<br>(36.8)	75<br>(1.2)	883<br>(14.1)	1359<br>(21.7)	229<br>(3.7)	450<br>(7.2)	713<br>(11.4)	14<br>(0.2)	88<br>(1.4)	103<br>(1.6)	6271<br>(100)

AB Agriculture, hunting and forestry, fishing
CD Mining and quarrying, manufacturing
E Electricity, gas and water supply
F Construction
G Wholesale and retail trade
H Hotels and restaurants
I Transport, storage, and communication
K Real estate, renting, and business activities
L Public administration and defence; compulsory social security
M Education
N Health and social work
O Other community, social, and personal service activities
