# Supplementary material for: Promoting prevention with economic arguments – The case of Finnish occupational health services
Source: BMC Public Health. 2008 Apr 22;8:130. doi: 10.1186/1471-2458-8-130 (PMC2386811; doi:10.1186/1471-2458-8-130)
Supplement: Additional file 2 — Correlations of variables. [file 1471-2458-8-130-S2.pdf]

Additional file 2 – Correlations of variables

	Age of company	Equity ratio in 1999	Log number of employees in 2001	Log turnover in 1999	Log (minutes per employee for prevention in 1999 +1))	Log (minutes per employee for prevention in 1997 +1)	Costs of prevention per turnover in 1999	Costs of prevention per turnover in 1997
Age of company	1.0000							
Equity ratio in 1999	0.0961	1.0000						
Log number of employees in 2001	0.1141	0.0566	1.0000					
Log turnover in 1999	0.1439	0.0835	0.7992	1.0000				
Log (minutes per employee for prevention in 1999 +1)	0.1139	0.0946	0.3612	0.3809	1.0000			
Log (minutes per employee for prevention in 1997 +1)	0.1369	0.0851	0.3682	0.3930	0.5368	1.0000		
Costs of prevention per turnover in 1999	0.0112	0.0068	0.1226	-0.0402	0.4030	0.1798	1.0000	
Costs of prevention per turnover in 1997	0.0300	0.0100	0.1773	0.0193	0.2680	0.5098	0.3055	1.0000
